# Effect of ammonium stress on phosphorus solubilization of a novel marine mangrove microorganism *Bacillus aryabhattai* NM1-A2 as revealed by integrated omics analysis

**DOI:** 10.1186/s12864-023-09559-z

**Published:** 2023-09-18

**Authors:** Zhaomei Lu, Sheng He, Muhammad Kashif, Zufan Zhang, Shuming Mo, Guijiao Su, Linfang Du, Chengjian Jiang

**Affiliations:** 1 State Key Laboratory for Conservation and Utilization of Subtropical Agro-bioresources, Guangxi Research Center for Microbial and Enzyme Engineering Technology, College of Life Science and Technology, Guangxi University, Nanning, 530004 China; 2 Guangxi Key Laboratory for Green Processing of Sugar Resources, College of Biological and Chemical Engineering, Guangxi University of Science and Technology, Liuzhou, 545006 China; 3 Key Laboratory of Bio-resources and Eco-environment of the Ministry of Education, College of Life Sciences, Sichuan University, Chengdu, 610064 China; 4 Guangxi Key Laboratory of Birth Defects Research and Prevention, Guangxi Key Laboratory of Reproductive Health and Birth Defect prevention, Guangxi Zhuang Autonomous Region Women and Children Health Care Hospital, Nanning, 530033 China

**Keywords:** Phosphorus solubilization, Ammonium stress, Ammonium assimilation, Omics analysis, Marine microorganisms

## Abstract

**Background:**

Phosphorus is one of the essential nutrients for plant growth. Phosphate-solubilizing microorganisms (PSMs) can alleviate available P deficiency and enhance plant growth in an eco-friendly way. Although ammonium toxicity is widespread, there is little understanding about the effect of ammonium stress on phosphorus solubilization (PS) of PSMs.

**Results:**

In this study, seven PSMs were isolated from mangrove sediments. The soluble phosphate concentration in culture supernatant of *Bacillus aryabhattai* NM1-A2 reached a maximum of 196.96 mg/L at 250 mM (NH_4_)_2_SO_4_. Whole-genome analysis showed that *B. aryabhattai* NM1-A2 contained various genes related to ammonium transporter (*amt*), ammonium assimilation (i.e., *gdhA*, *gltB*, and *gltD*), organic acid synthesis (i.e., *ackA*, *fdhD*, and *idh*), and phosphate transport (i.e., *pstB* and *pstS*). Transcriptome data showed that the expression levels of *amt*, *gltB*, *gltD*, *ackA* and *idh* were downregulated, while *gdhA* and *fdhD* were upregulated. The inhibition of ammonium transporter and glutamine synthetase/glutamate synthase (GS/GOGAT) pathway contributed to reducing energy loss. For ammonium assimilation under ammonium stress, accompanied by protons efflux, the glutamate dehydrogenase pathway was the main approach. More 2-oxoglutarate (2-OG) was induced to provide abundant carbon skeletons. The downregulation of formate dehydrogenase and high glycolytic rate resulted in the accumulation of formic acid and acetic acid, which played key roles in PS under ammonium stress.

**Conclusions:**

The accumulation of 2-OG and the inhibition of GS/GOGAT pathway played a key role in ammonium detoxification. The secretion of protons, formic acid and acetic acid was related to PS. Our work provides new insights into the PS mechanism, which will provide theoretical guidance for the application of PSMs.

**Supplementary Information:**

The online version contains supplementary material available at 10.1186/s12864-023-09559-z.

## Background

Phosphorus (P) is essential to mangrove ecosystems, which is implicated as one of the most limiting nutrients for plant growth [1, 2]. Despite the high amount of total P in the soil, most of the P cannot be absorbed and utilized by plants [1, 3, 4]. Chemical fertilizers have been widely used to satisfy the demand for P to promote plant productivity [4]. However, the long-term application of chemical fertilizers has led to a series of problems limiting agricultural sustainable development, such as soil nutrient imbalance and water eutrophication [3, 4]. Consequently, it is urgent to find an eco-friendly and economically viable manner to maintain a continuous supply of P in plants. Phosphorus solubilization (PS) is a significant biochemical process in sediments, and plays important roles in alleviating P limitations of mangrove ecosystems [5]. Several microorganisms can transform insoluble inorganic P into soluble forms available to plants, which are known as phosphate-solubilizing microorganisms (PSMs) [1, 6]. Furthermore, PSMs can not only release the accumulated P left by P fertilizer but also promote plant growth without environmental hazards [1, 6]. PSMs mainly include phosphate-solubilizing bacteria (PSB) and phosphate-solubilizing fungi (PSF). PSB include various strains from the genera *Pseudomonas*, *Bacillus*, *Agrobacterium Azotobacter*, *Xanthomonas*, and *Enterobacter* [7, 8]. PSF include strains of *Penicillium*, *Mucor*, *Aspergillus*, *Pichia*, and *Chaetomium* [8, 9]. These microorganisms can serve as biofertilizers that improve the agricultural yield [10].

The secretion of low molecular weight organic acids is considered to be the principal mechanism for mineral phosphate dissolution [3, 11]. These organic acids can liberate P either by lowering the pH or chelating the cations bound to phosphate [10]. Gluconic acid produced by the direct oxidation of glucose is usually regarded as the main organic acid in the solubilization of mineral phosphate [3]. Accumulating evidence suggested that intermediates in the tricarboxylic acid cycle (TCA cycle), such as succinic acid, malic acid, and citric acid, were also the main organic acids secreted during the process of PS [11]. Other mechanisms such as the production of inorganic acids and chelating substances, are less effective than organic acids in releasing P [8]. PS conducted by PSMs is a complicated process. The efficiency of PS is affected by many factors such as nutrition, pH, and oxygen concentration [12, 13]. Since PSMs are heterotrophic, it is generally accepted that carbon (C) and nitrogen (N) are two of the limiting factors for the growth of PSMs [12]. It was reported that easily utilizable C source was deemed as a contributing factor to increase the PS efficiency of PSMs [14]. Besides, ammonium (NH_4_^+^) was also the best N source for PS, among various nutrients used by microorganisms [9].

NH_4_^+^ is a fundamental substrate for nucleic acid and amino acid synthesis [15, 16]. It has been proved that NH_4_^+^ played a positive role as a stress signal [16]. For instance, NH_4_^+^ nutrition enhanced salt stress resistance in citrus plants due to the induction of the antioxidant cellular machinery [17]. Moreover, increased alkaline phosphatase activity led to the solubilization of P under high NH_4_^+^ stress [18]. However, excessive NH_4_^+^ damages the growth of the living organism, which is widely known as NH_4_^+^ toxicity [19]. NH_4_^+^ toxicity is a significant ecological and agricultural issue [15]. Several hypotheses for the cause of toxicity have been proposed, including the futile transmembrane NH_4_^+^ cycle, the lack of inorganic cations and organic acids, the impairment of hormone homeostasis, and the disorder of pH regulation [20]. A previous study found that supplemented succinate effectively alleviated NH_4_^+^ toxicity [20]. Therefore, it was assumed that the production of organic acids in PS process performed a significant role in resisting NH_4_^+^ toxicity. Until now, few studies have focused on the influence of NH_4_^+^ stress on releasing precipitated inorganic P.

In our current work, an efficient PSM *Bacillus aryabhattai* NM1-A2 was isolated from the marine mangrove sediment. The effects of different C sources, N sources, C/N, (NH_4_)_2_SO_4_ concentrations, NaCl concentrations and initial pH of solution on the solubilization of insoluble phosphate were investigated to determine its PS characteristics. Then, the effect of NH_4_^+^ stress on PS was explored at the level of genome, transcriptome, and metabolite. Our work demonstrates the scientific basis for the application of PSMs, and it will promote environment-friendly agricultural development.

## Results

### Isolation and identification of PSMs

**Fig. 1:**
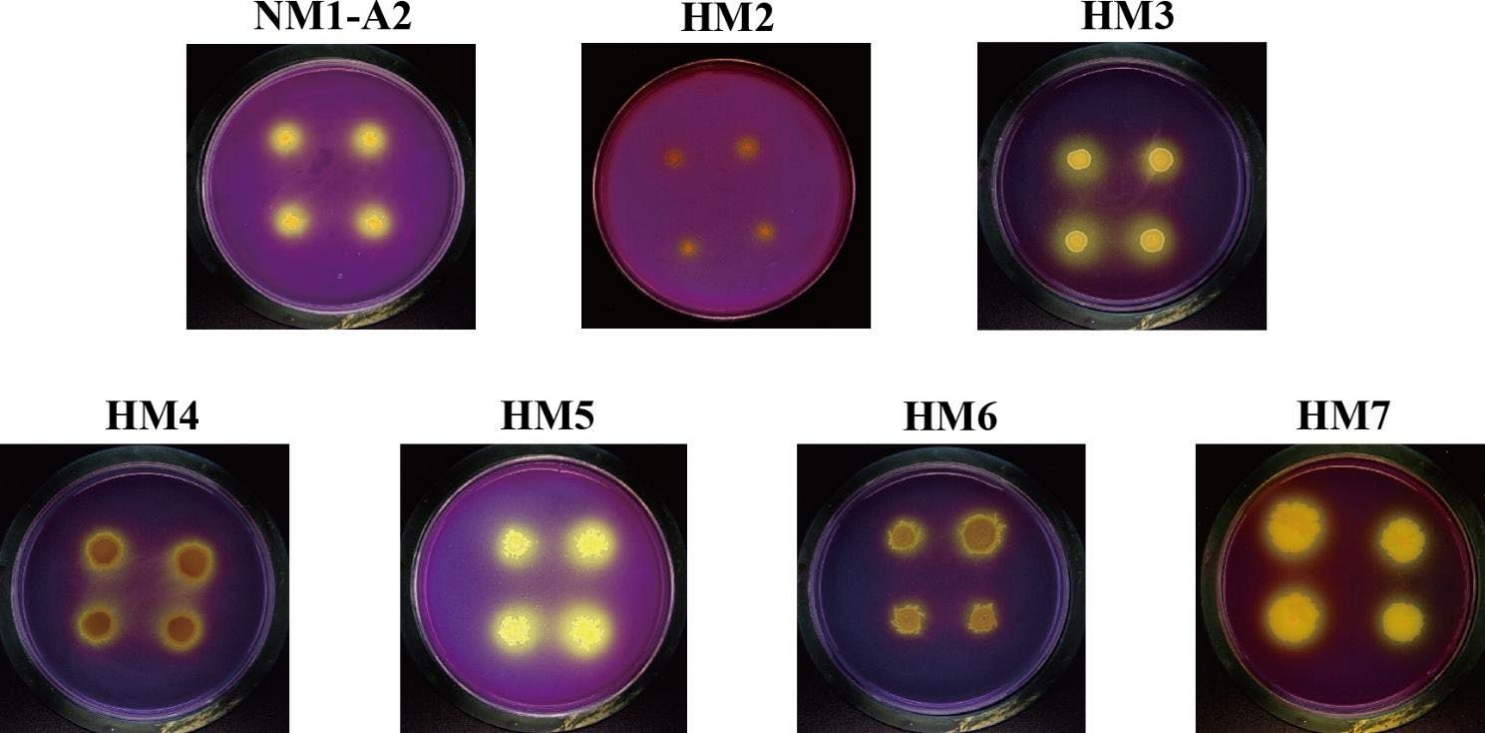
Isolation of phosphorus-solubilizing microorganisms (PSMs). Seven PSMs screened in this study were labeled as NM1-A2, HM2, HM3, HM4, HM5, HM6 and HM7, respectively

Seven PSMs were selectively obtained from the mangrove sediments based on the obvious phosphate-solubilizing halos around colonies on SRSM solid medium (the enrichment medium invented by Sundara Rao and Sinha specifically for the isolation of PSMs) (Fig. 1). The bromocresol purple indicator on the SRSM medium changed from purple to yellow (Fig. 1), which was a sign of pH drop. Thus, the seven PSMs might dissolve mineral phosphate by secreting organic acids or expelling protons.

**Fig. 2:**
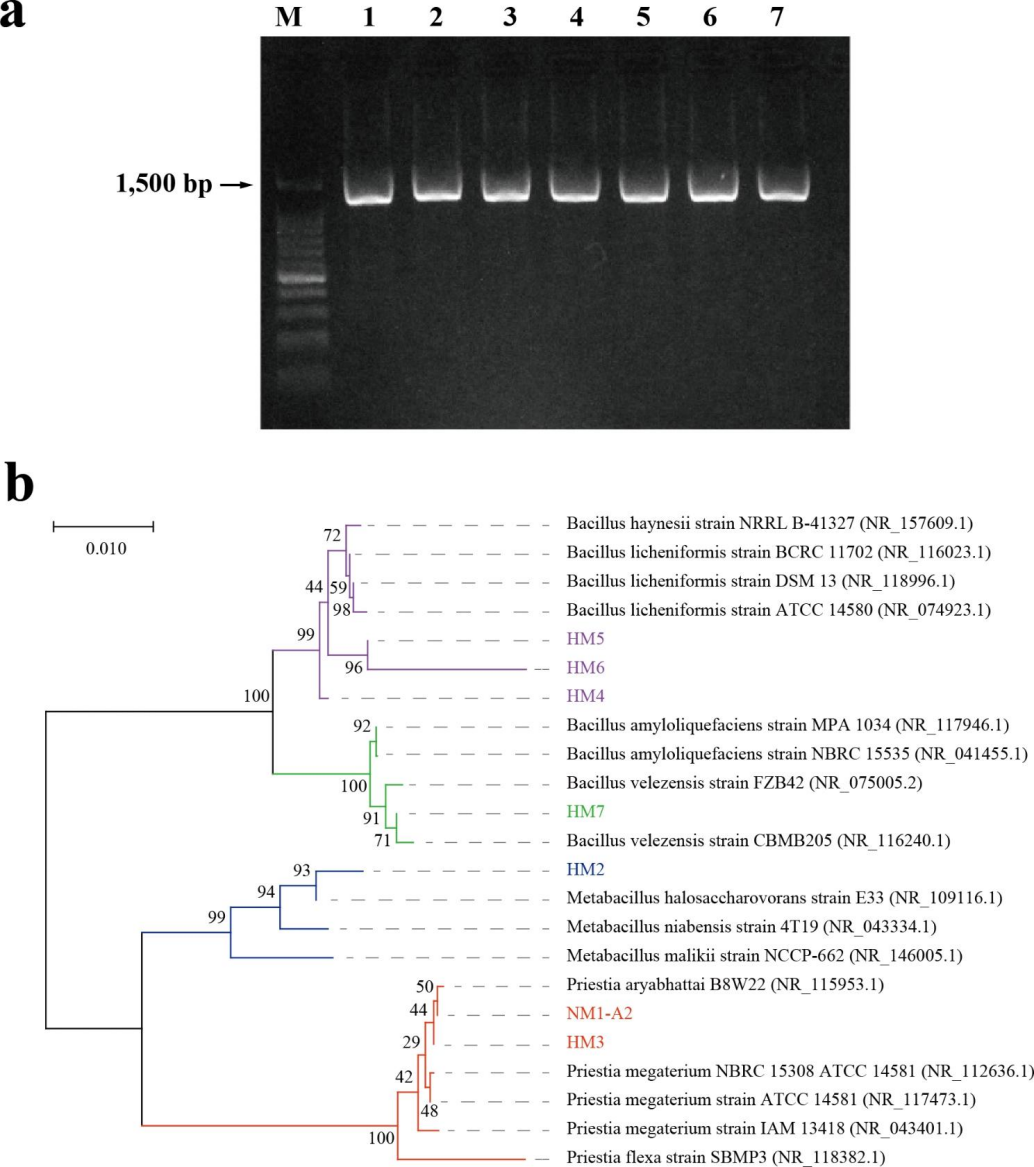
Identification of seven PSMs screened in this study. (**a**) Evaluation of 16 S rDNA PCR products by agarose gel electrophoresis. (**b**) Neighbour-joining phylogenetic tree based on 16 S rDNA. Bootstrap analyses were made with 1000 cycles. M, 1, 2, 3, 4, 5, 6 and 7 indicate Marker, NM1-A2, HM2, HM3, HM4, HM5, HM6 and HM7, respectively

We performed the PCR amplifications using specific primers [21] (Additional file 1: Table S1) for 16 S rDNA. 16 S rDNA sequences were subjected to agarose gel electrophoresis, and bright bands were detected at 1, 500 bp (Fig. 2a). The results showed that the seven PSMs were pure strains. The nucleotide sequences of 16 S rDNA were analyzed with the BLASTn search algorithm and aligned to their nearest neighbors (Table S2). A phylogenetic tree was created based on 16 S rDNA nucleotide sequences of the isolates and their nearest neighbors (Fig. 2b). It was found that NM1-A2 and HM3 were most closely related to *B. aryabhattai*. HM5 and HM6 were most closely related to *B. licheniformis*. HM2, HM4, and HM7 were most closely related to *Metabacillus halosaccharovorans*, *B. haynesii*, and *B. velezensis*, respectively.

### Determination of the soluble phosphate concentration in culture supernatant

The soluble phosphate concentration in culture supernatant of seven PSMs varied from 22.65 mg/L to 139.46 mg/L. Except for HM2 and HM7, the concentration of soluble phosphate decreased as time progressed. The soluble phosphate concentration in culture supernatant of *B. aryabhattai* NM1-A2 reached 139.46 mg/L at 48 h, which was much higher than those of other strains (Table 1).

**Table 1:** The concentration of soluble phosphate in culture supernatant when strain was cultured in SRSM medium for 12 h

Strain	Soluble phosphate concentration (mg/L)			
	48 h	72 h	96 h	120 h
NM1-A2	139.46 ± 0.42 a	95.00 ± 1.35 a	54.28 ± 0.30 b	32.12 ± 1.04 bc
HM2	37.18 ± 3.96 c	40.29 ± 2.06 d	34.65 ± 0.93 c	30.76 ± 3.13 bc
HM3	116.19 ± 2.99 b	97.19 ± 1.53 a	74.69 ± 4.99 a	35.33 ± 2.49 b
HM4	85.14 ± 5.28 c	35.48 ± 3.16 d	25.52 ± 1.96 c	18.62 ± 4.31 d
HM5	85.04 ± 2.62 c	65.36 ± 4.07 bc	25.03 ± 0.95 c	26.44 ± 1.30 bcd
HM6	62.88 ± 9.08 d	57.59 ± 5.93 c	29.35 ± 15.77 c	22.65 ± 3.07 cd
HM7	64.39 ± 8.07 d	72.60 ± 5.74 b	62.06 ± 1.60 ab	57.68 ± 5.47 a

**Fig. 3:**
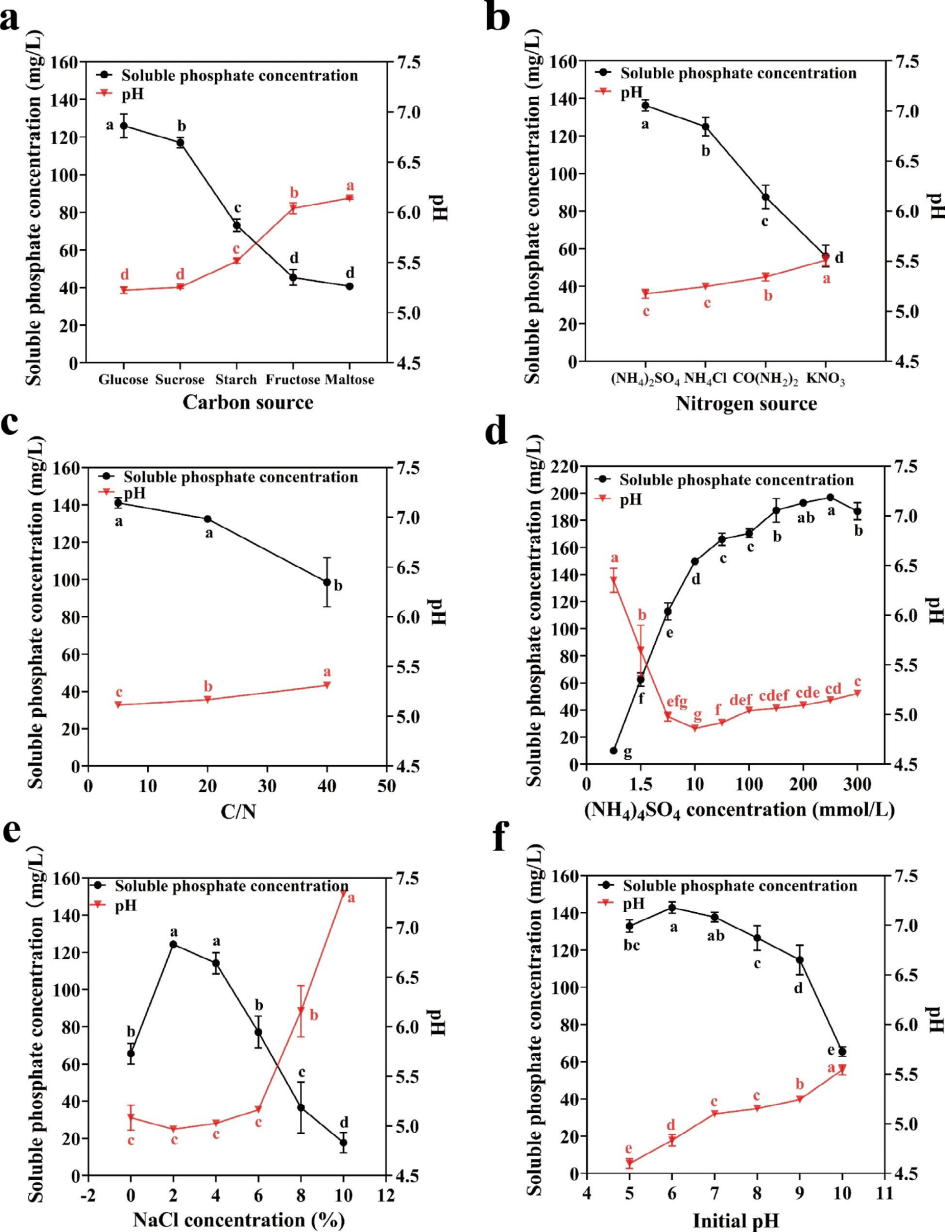
(**a**) Effects of carbon sources, (**b**) nitrogen sources, (**c**) C/N, (**d**) (NH_4_)_2_SO_4_ concentrations, (**e**) NaCl concentrations, and (**f**) initial pH on the solubilization of tricalcium phosphate by *B. aryabhattai* NM1-A2. The least significant difference test based on the analysis of variance model was used to calculate statistical significance, as expressed in means ± standard error. And values significantly different from those of the control were represented by different lowercase letters

The PS process of *B. aryabhattai* NM1-A2 on SRSM liquid medium containing different components was examined to better understand its PS characteristics. It was found that the solubilization of tricalcium phosphate (TCP) in the SRSM liquid medium was accompanied with the decrease of pH after 12 h (Fig. 3). The correlation analysis showed a significant negative correlation between the soluble phosphate concentration and pH in culture supernatant of *B. aryabhattai* NM1-A2 (Table 2). The results suggested that the secretion of organic acids or the release of protons might lead to a decrease in pH, which might be one of the PS mechanisms. The highest concentration of soluble phosphate reached 124.24 mg/L when *B. aryabhattai* NM1-A2 was cultivated with glucose as the sole C source, followed by sucrose, starch, fructose, and maltose (117, 73.07, 45.38 and 40.68 mg/L, *p* < 0.05) (Fig. 3a). As shown in Fig. 3b, the optimum N source for PS was (NH_4_)_2_SO_4_ (136.29 mg/L), followed by NH_4_Cl, CO(NH_2_)_2_, KNO_3_ (124.99, 87.51, and 56.08 mg/L, *p* < 0.05). The soluble phosphate concentration in culture supernatant of *B. aryabhattai* NM1-A2 decreased rapidly with the increase in C/N ratio in the SRSM lipid medium (Fig. 3c). In addition, the efficiency of PS was affected by the concentration of (NH_4_)_2_SO_4_. The highest soluble phosphate concentration of 196.96 mg/L was dissolved at 250 mM of (NH_4_)_2_SO_4_ concentration. in the range from 1 mM to 300 mM (Fig. 3d). And the production of soluble P could reach the highest when NaCl concentration was 2% in the range from 0 to 10% (Fig. 3e). Subsequently, the soluble phosphate concentration decreased continuously with the increase of NaCl concentration (Fig. 3e), which indicated that the process of PS was inhibited under salt stress. The change of pH value directly affected the survival and metabolism of microorganisms. As shown in Fig. 3f, B. *aryabhattai* NM1-A2 grew normally in the range of pH 5.0–10.0. The highest soluble phosphate concentration (142.79 mg/L) was detected when the initial pH value was 6.0, followed by 7.0, 5.0, 8.0, 9.0 and 10.0 (137.65, 132.87, 126.504, 114.74, and 65.37 mg/L).

**Table 2:** Correlation coefficient between soluble phosphate concentration and pH while *Bacillus aryabhattai* NM1-A2 was cultured under different conditions

Factor	Pearson correlation coefficient (r)	*p*-value
Carbon (C) sources	-0.958	*p* < 0.01
N sources	-0.968	*p* < 0.01
C/N	-0.924	*P* < 0.01
NaCl concentrations	-0.831	*P* < 0.01
pH	-0.779	*P* < 0.01
(NH_4_)_2_SO_4_ concentrations	-0.902	*P* < 0.01

### Effect of NH_4_^+^ stress on the types and concentrations of organic acids

**Fig. 4:**
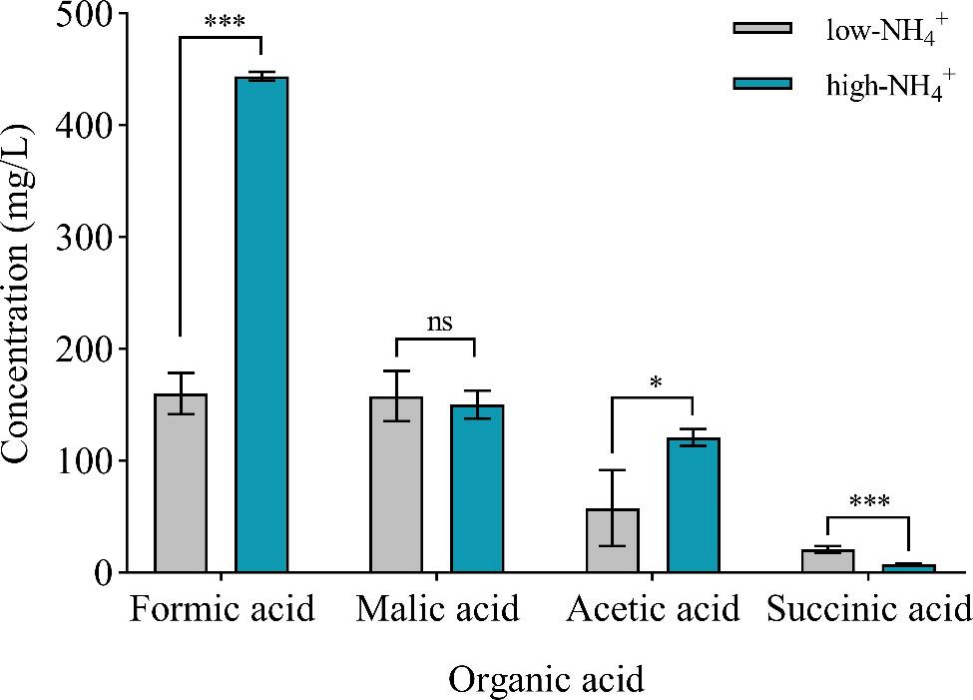
Kinds and concentrations of organic acids in the supernatants of *B. aryabhattai* NM1-A2 in the low- and high-NH_4_^+^ groups. Two independent-sample t-test was used to determine differences in organic acid concentration between the low- and high-NH_4_^+^ group. And values significantly different from those of the control were represented by asterisks, which “∗” and “∗∗∗” indicated *p* < 0.05 and *p* < 0.001, respectively, while “ns” indicated no significant difference

As we know, PS process of PSMs was associated with the release of low molecular weight organic acids [3, 11]. High performance liquid chromatography (HPLC) analysis showed the presence of four organic acids in culture supernatant, namely formic acid, malic acid, acetic acid and succinic acid, while lactic acid and citric acid were not detected (Fig. S1 and Table S3). Among the four organic acids, formic acid showed the highest concentration, followed by malic acid, acetic acid and succinic acid (Fig. 4). As shown in Fig. 4, the concentrations of formic acid and acetic acid in the high-NH_4_^+^ group were 443.7 and 120.5 mg/L at 12 h, respectively. These concentrations were significantly higher than those in the low-NH_4_^+^ group (160.1 and 57.3 mg/L at 12 h, *p* < 0.001). As shown in Fig. 4, the concentrations of succinic acid in the high-NH_4_^+^ group was 7.5 mg/L at 12 h, which was lower than that in the low-NH_4_^+^ group (20.8 mg/L, *p* < 0.05). However, there was no significant difference in the concentration of malic acid between the low- and high-NH_4_^+^ group (Fig. 4). The increase of soluble phosphate concentration in culture supernatant of *B. aryabhattai* NM1-A2 could be potentially due to the production of organic acids. And the types and concentrations of secreted organic acids were influenced by (NH_4_)_2_SO_4_ concentration.

### Whole-genome assembly and annotation of *B. aryabhattai* NM1-A2

NH_4_^+^ was a preferred N source for most bacteria [2]. The high-affinity NH_4_^+^ transporters AMTs (encoded by *amt*) were used to absorb NH_4_^+^ at low NH_4_^+^ concentrations, while some low-affinity transporters were responsible for NH_4_^+^ at high NH_4_^+^ concentrations [22]. The whole-genome of *B. aryabhattai* NM1-A2 (Table S4) contained genes coding for glutamine synthetase (GS, encoded by *glnA*), glutamate synthase (GOGAT, encoded by *gltBD*) and glutamate dehydrogenase (GDH, encoded by *gdhA*), which were key enzymes for NH_4_^+^ assimilation [23].

In addition, there were many types of PS-related genes, mainly including genes involved in organic acid synthesis and the phosphate regulatory system (Table S4). Inorganic phosphate (Pi) was mainly obtained through two independent uptake systems, inorganic phosphorus transporter (Pit) and phosphorus specific transporter (Pst) [24]. The Pst system was a typical ABC transporter composed of PstS, PstC, PstA, PstB and PhoU [24, 25]. Pi was transported by the Pst system under P starvation [25, 26]. *B. aryabhattai* NM1-A2 also had histidine kinase PhoR, response regulator PhoB for induction of PHO regulon under P-limiting conditions [27]. The main enzymes involved in TCA cycle and organic acid synthesis were found to be: isocitrate lyase, formate dehydrogenase, acetate kinase and isocitrate dehydrogenase, fumarate hydratase, and malate dehydrogenase, encoded by *aceA*, *fdhD*, *ackA*, *idh*, *fumC* and *mdh*, respectively.

### Transcriptome sequencing of *B. aryabhattai* NM1-A2 and annotation analysis of DEGs

**Fig. 5:**
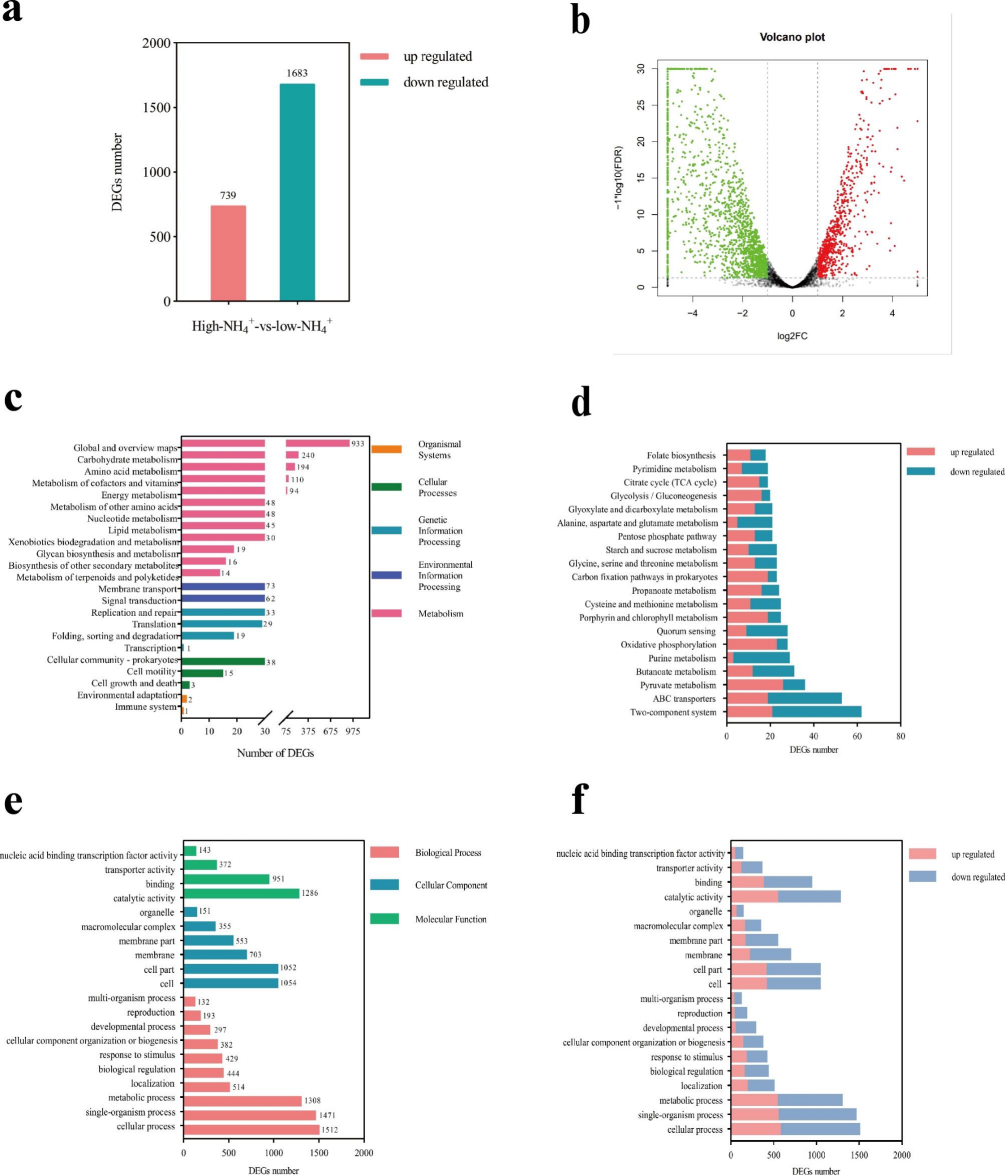
Effect of NH_4_^+^ stress on *B. aryabhattai* NM1-A2 gene transcription. (**a**) Numbers of upregulated and downregulated differentially expressed genes (DEGs). (**b**) Volcano plot presentation of overall scattered DEGs. (**c**) KEGG enrichment pathway of DEGs [1]. (**d**) Upregulated or downregulated expressions of DEGs of the top 20 most enriched KEGG pathways [1]. (**e**) GO annotation of DEGs. (**f**) Upregulated or downregulated expressions of the top 20 most enriched GO terms

Transcriptome data results showed 2422 significantly differentially expressed genes (DEGs) between the low- and high-NH_4_^+^ groups. A total of 739 significantly upregulated genes and 1683 significantly downregulated genes were detected (Fig. 5a and b). KEGG enrichment results showed that the DEGs were mainly enriched in carbohydrate metabolism, amino acid metabolism, metabolism of cofactors and vitamins, and energy metabolism (Fig. 5c). C and N metabolism played an important role in response to NH_4_^+^ stress [22]. Moreover, the numbers of upregulated or downregulated DEGs of the top 20 most enriched KEGG pathways were displayed (Fig. 5d). The genes related to pyruvate metabolism, oxidative phosphorylation, glycolysis, and TCA cycle were mostly upregulated. The GO enrichment analysis of DEGs between the low- and high-NH_4_^+^ groups (Fig. 5e and f) showed that the main enriched biological processes were cellular process, single organization and metabolic processes. The main enriched cellular components were cell and cell part. The main enriched functions were binding, catalytic activity, and transporter activity. These results indicated that catalytic and transport activities in *B. aryabhattai* NM1-A2 were significantly affected by high NH_4_^+^ stress.

**Fig. 6:**
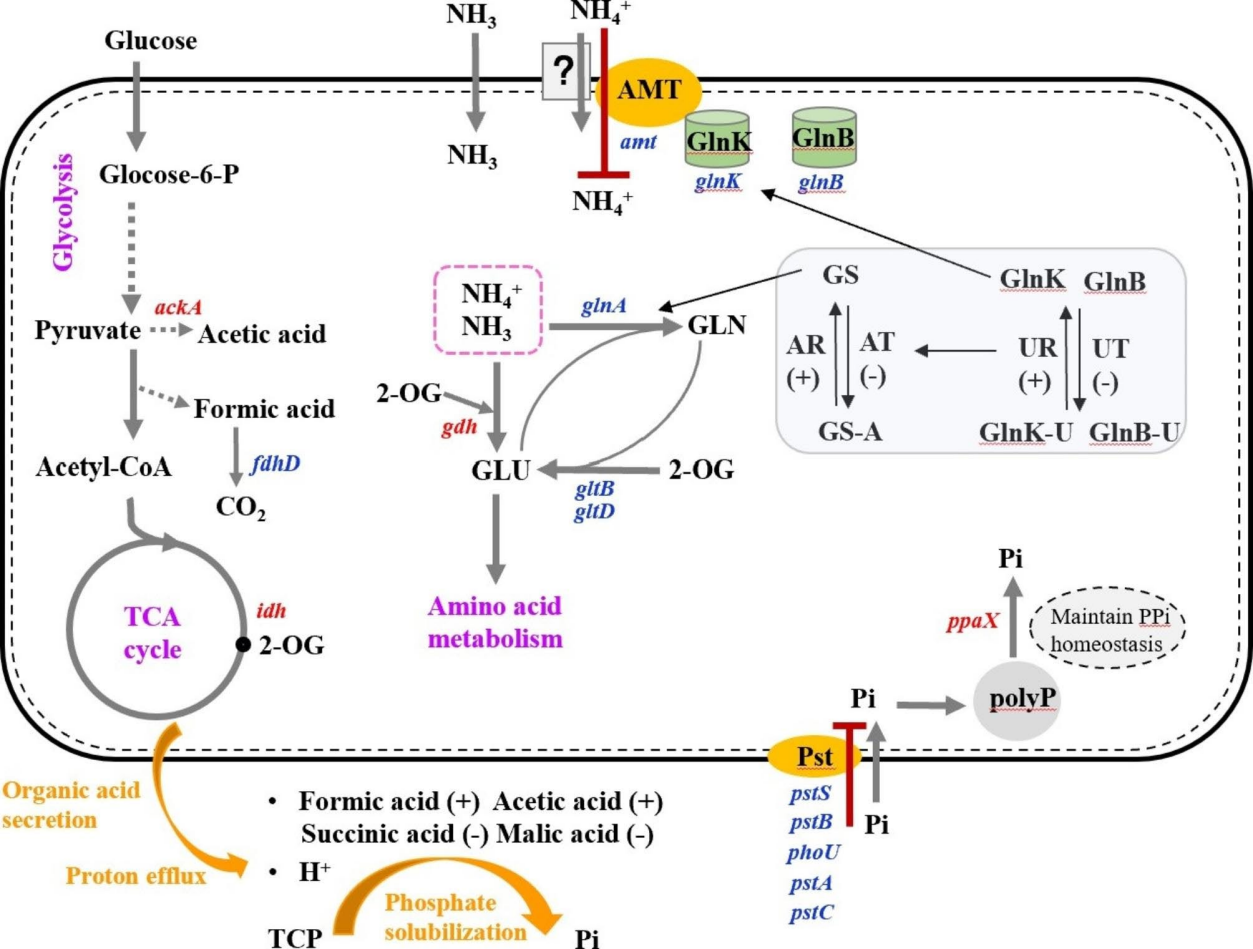
NH_4_^+^ tolerance mechanism of *B. aryabhattai* NM1-A2. “+” indicated that enzyme activity or substance content increases under NH_4_^+^ stress. “−“ indicated that enzyme activity or substance content reduces under NH_4_^+^ stress. Red letters indicated downregulation genes, and navy letters indicated upregulation genes. Purple letters indicated pathways, and orange ovals indicated transporters. Solid arrow corresponded to direct reaction, and dotted arrow corresponded to indirect reaction. The red line indicated that the process is inhibited. 2-OG, TCP, Pi, polyP, Pst, AMT, GLU, GLN, and GS indicated 2-oxoglutarate, tricalcium phosphate, inorganic phosphorus, polyphosphate, phosphorus specific transporter, ammonium transporter, glutamate, glutamine, and glutamine synthetase, respectively

The transcriptome data showed that the expression level of *amt* was downregulated by 3.52 fold, which indicated that the NH_4_^+^ transported by AMT was reduced (Table 3; Fig. 6). NH_4_^+^ assimilation was regulated by two P_II_ proteins (GlnB and GlnK) [28]. GlnB and GlnK could be reversibly uridylylated by uridilyl-transferase/uridylyl-removase (UT/UR), controlling the bifunctional enzyme adenylyl-transferase/adenylyl-removase (AT/AR), which could reversibly adenylylate (inactivate) or deadenylylate (activate) GS [28, 29]. GlnK was commonly cotranscribed with the NH_4_^+^ transporter AMT and reversibly inhibited AMT’s transport activity [29]. The expression levels of *glnB* and *glnK* were downregulated by 2.779 and 2.323 folds, respectively (Table 3). AR enzymatic activity was promoted through the deuridylylation of GlnK-U and GlnB-U to rapidly inhibited the activity of GS. Then, GlnK binded to AMT to form a complex to inhibit NH_4_^+^ uptake.

**Table 3:** DEGs related to NH_4_^+^ assimilation, phosphate transport and metabolism in *B. aryabhattai* NM1-A2.

Locus	Genes symbol	Gene description	Log_2_(B/A)
K8Z47_RS03380	*amt*	Ammonium transporter	-3.518
K8Z47_RS11985	*gdhA*	Glutamate dehydrogenase	1.306
K8Z47_RS20175	*glnA*	Glutamine synthetase, type I	-0.765
K8Z47_RS21810	*gltD*	Glutamate synthase [NADPH] small chain	-3.377
K8Z47_RS10260	*gltB*	Glutamate synthase (NADPH) large chain	-1.425
K8Z47_RS25960	*phoR*	Phosphate regulon sensor histidine kinase PhoR	-5.080
K8Z47_RS06210	*phoB*	Phosphate regulon response regulator PhoB	-2.901
K8Z47_RS22250	*phoU*	Phosphate transport system regulatory protein PhoU	-4.023
K8Z47_RS22810	*pstS*	Phosphate-binding protein PstS 1 precursor	-5.293
K8Z47_RS22805	*pstC*	Phosphate ABC transporter, permease protein PstC	-2.305
K8Z47_RS22800	*pstA*	Phosphate transport system permease protein PstA	-2.319
K8Z47_RS22255	*pstB*	Phosphate ABC transporter, ATP-binding protein PstB	-5.707
K8Z47_RS25095	*ppaX*	Pyrophosphatase PpaX	2.062
K8Z47_RS23660	*ackA*	Acetate kinase	1.305
K8Z47_RS24905	*fdhD*	Formate dehydrogenase accessory protein	-1.583
K8Z47_RS23540	*icd*	Isocitrate dehydrogenase [NADP]	1.256
K8Z47_RS03260	*aceA*	Isocitrate lyase	-2.936
K8Z47_RS01990	*fumA*	Fumarate hydratase, class I	3.451
K8Z47_RS23535	*mdh*	Malate dehydrogenase	1.240
K8Z47_RS05365	*glnK*	P_II_ family nitrogen regulator	-0.908
K8Z47_RS03375	*glnB*	P family nitrogen regulator	-2.7789

The GS-GOGAT cycle was the most crucial pathway for primary N assimilation [30, 31]. NH_4_^+^ and glutamate (GLU) to form glutamine (GLN) under the catalysis of GS (GlnA) [29]. The large subunit (GltB) and small subunit (GltD) of GOGAT complete the metabolic pathway of GLU regeneration from GLN and 2-oxoglutarate (2-OG) [29]. The expression levels of *gltB*, and *gltD* were downregulated by 1.425, and 3.377 folds, respectively (Table 3), which revealed that the GS − GOGAT pathway was not the main approach for NH_4_^+^ assimilation in *B. aryabhattai* NM1-A2. GDH has been reported to incorporate excess NH_4_^+^ into GLU when exposed to NH_4_^+^ stress [23]. The expression level of *gdhA* was upregulated in the high-NH_4_^+^ group compared with that in the low-NH_4_^+^ group (Table 3). Therefore, the GDH pathway played a critical role in responding to NH_4_^+^ stress (Fig. 6). The assimilation of NH_4_^+^ was accompanied by the release of protons, which was one of the mechanisms of PS (Fig. 6).

The production of organic acids was the main mechanism for solubilizing insoluble P [9]. The transcriptome data showed that the expression level of *ackA* was upregulated, wheras *fdhD* was downregulated (Table 3; Fig. 6). This might lead to increased concentrations of acetic acid and formic acid in high-NH_4_^+^ group (Fig. 4). Therefore, formic acid and acetic acid played an important role in PS process of *B. aryabhattai* NM1-A2 under NH_4_^+^ stress (Fig. 6). In addition, *idh* was also upregulated by 1.256 fold. More 2-OG was produced under NH_4_^+^ stress, which provided sufficient C skeletons for the subsequent amino acid synthesis. The expression level of *aceA* was downregulated (Table 3), which might be the reason behind the decrease in succinic acid in the high-NH_4_^+^ group (Fig. 4). *fumC* and *mdh* were upregulated in the high-NH_4_^+^ group compared with those in the low-NH_4_^+^ group (Table 3). The catabolism of malic acid was greater than the synthesis, which led to the decrease in malic acid concentration under NH_4_^+^ stress.

The Pst system played an important role in transporting inorganic P under conditions of P limitation [27]. PstS and PstB were the key proteins in Pst system [24]. Genes related to the Pst system and PhoR were downregulated under NH_4_^+^ stress, especially *pstS* and *pstB* (downregulated by 5.29 and 5.71 folds). As shown in the Fig. 3d, the content of soluble phosphate in the high-NH_4_^+^ group (196.96 mg/L) was significantly higher than that in the low-NH_4_^+^ group (62.5 mg/L, *p* < 0.001). As a result, the Pst transporter was inhibited. The signal was transmitted from the Pst transporter to PhoR through PhoU, and inhibited the kinase function of PhoR, resulting in dephosphorylation of PhoB [27]. When Pi was abundant, excess P was stored as polyphosphate (PolyP) [32]. PolyP could be separated into two groups, pyrophosphates (two phosphate residues) and high molecular weight PolyP [33]. Pyrophosphates (PPi) was a metabolic product of biosynthetic reactions, and pyrophosphatase (PPaX) catalyzed the hydrolysis of PPi [33]. *ppaX* was upregulated in high-NH_4_^+^ group, which could maintain cellular PPi homeostasis and provide energy for the PPi-generating biosynthetic reactions (Table 3; Fig. 6).

### RT-qPCR of genes involved in PS of *B. aryabhattai* NM1-A2 under NH_4_^+^ stress

Quantitative real-time polymerase chain reaction (RT-qPCR). According to the abovemetioned results, eight genes (*gltD*, *ackA*, *pstB*, *ppaX*, *pstB*, *amtB*, *phoR*, and *glnA*) were selected for RT-qPCR analysis. The expression levels of *ackA* and *ppaX* were upregulated in the high-NH_4_^+^ group, whereas *gltD*, *pstB*, *pstB*, *amtB*, *phoR*, and *glnA* were downregulated (Fig. S2). The expression trends of eight genes were consistent with the RNA-seq data, which confirmed the reliability of the transcriptomic data.

## Discussion

P is an essential nutrient element for plant growth and development [1, 2]. PSMs can promote the availability and absorption of P in soil [8]. The application of PSMs plays a very important role in the sustainable development of agriculture [1, 6].

In this study, TCP was selected as the sole P source on SRSM medium because it represented the vast majority of insoluble P [34]. Based on cultivation and observation, seven PSMs were screened from marine mangrove sediments (Fig. 1). According to 16 S rDNA sequence analysis, these strains were confirmed as members of *Bacillus* genus. Several studies have shown that *Pseudomonas*, *Bacillus* and *Rhizobia* were effective phosphate solubilizers [7, 8]. *Bacillus* spp. were preferred on account of lasting vitality [35]. The soluble phosphate concentrations in culture supernatant of these strains were evaluated by molybdenum blue colorimetric method [21]. Among them, the soluble phosphate concentration in culture supernatant of *B. aryabhattai* NM1-A2 reached a maximum of 139.46 mg/L at 48 h (Table 1). In addition, the soluble phosphate concentration in culture supernatant decreased as time progressed, which could probably be attributed to the utilization of P by bacteria [36]. *Bacillus aryabhattai* was widely distributed in various habitats owing to its strong stress resistance, and had great potential in nitrogen fixation, PS and the production of indole-3-acetic acid [36–38]. However, few studies have revealed the genomic potential of *Bacillus aryabhattai* on PS.

PSMs are heterotrophic microorganisms. The PS efficiency of PSMs depends on the provision of suitable C and N in a large extent [12]. Scervino et al. reported that glucose and (NH_4_)_2_SO_4_-based media showed the highest PS values [12], which was consistent with the results of this study (Fig. 3a and b). Glucose was the preferred C source for most bacterial growth [14]. However, Li’s report [6] showed that there was no correlation between cell growth and soluble P production [6]. Mineral phosphate could be solubilized by the direct oxidation of glucose to gluconic acid [8]. This condition might explain the maximum PS ability with glucose as C source (Fig. 3a). Among all N sources tested, the optimum N source for PS was (NH_4_)_2_SO_4_ (Fig. 3b). Bacterial growth was greatly enhanced with the increasing concentration of (NH_4_)_2_SO_4_, which led to the consumption of available P in the culture medium [6]. Interestingly, the soluble phosphate concentration in culture supernatant of *B. aryabhattai* NM1-A2 was continuously improved with the increase of (NH_4_)_2_SO_4_ concentration and reached the highest (196.96 mg/L) under 250 mM (NH_4_)_2_SO_4_ (Fig. 3d). NH_4_^+^ was the preferred N source for most bacteria, but it was toxic when reaching a certain concentration [37]. For example, *Nitrobacter winogradskyi* Nb-255 grew well under NH_4_^+^ concentrations below 25 mM, but it was inhibited under NH_4_^+^ concentrations higher than 35 mM [38]. It was reported that transcript degradation or gene repression was part of the NH_4_^+^-responsive stimulon [39]. Given its remarkable NH_4_^+^-tolerant survivability, the mechanism of PS and detoxification of *B. aryabhattai* NM1-A2 under NH_4_^+^ stress was revealed by multi-omics analysis.

Ammonium exists predominantly in the ionic form (NH_4_^+^) at neutral pH, and the minor gaseous species (NH_3_) can diffuse rapidly through the cell membrane. NH_4_^+^ could be transported into the cell via AMT under low ammonium conditions [40]. When bacteria encountered high concentration of NH_4_^+^, the passive diffusion of NH_3_ can provide enough N for optimal cell growth [41]. Transcriptome data showed that *amt* was downregulated, suggesting NH_4_^+^ transported via AMT was inhibited (Table 3). NH_4_^+^ assimilation was the main approach for NH_4_^+^ detoxification [22]. Under high-NH_4_^+^ condition, excess NH_4_^+^ could be assimilated into GLU mainly through GDH pathway, thereby reducing NH_4_^+^ accumulation (Table 3) [41, 42]. Downregulating the GS/GOGAT pathway under NH_4_^+^ stress was helpful to prevent the waste of energy (Table 3) [43]. Moreover, research shows that NH_4_^+^ assimilation by GS localized in the plastid rather than NH_4_^+^accumulation was a primary cause for toxicity [19]. In this study, GS enzyme activity of *B. aryabhattai* NM1-A2 was inhibited in the regulation of GlnB and GlnK under NH_4_^+^ stress, thereby preventing NH_4_^+^ toxicity (Fig. 6) [29]. Tian et al. reported that the sugar and starch contents of plants decreased under NH_4_^+^ treatment because of the depletion of C skeletons used for excess NH_4_^+^ assimilation [22]. In the present study, the overall upregulated glycolysis, pyruvate metabolism and TCA cycle could provide abundant C skeletons for excess NH_4_^+^ assimilation (Fig. 5d) [22]. Among them, the upregulated expression of *idh* indicated that more 2-OG was induced under NH_4_^+^ stress (Table 3) [44, 45]. It has been reported that supplemented 2-OG enhanced NH_4_^+^ assimilation [20]. That might be an important feature of *B. aryabhattai* NM1-A2 responded to NH_4_^+^ toxicity. The assimilation of NH_4_^+^ within microbial cells was accompanied by the release of protons resulting in the solubilization of P [3, 46].

The secretion of organic acids was an important phosphate-solubilizing way in PSMs, which could be produced from cells to facilitate the solubilization of P by supplying both protons and metal complexing organic acid anions [34]. Different PSMs would secrete the various types and concentrations of organic acids to solubilize P [3]. The production of succinic acid was related to PS activity of *Bacillus megaterium*, while oxalic acid and citric acid were the main organic acids released by *Pseudomonas* sp [3]. The secretion of organic acids was also influenced by the types and concentrations of P sources [47]. Arvind et al. found that the generation of formic acid was limited to the dissolution of TCP, and oxalic acid production to the solubilization of Mussoorie rock phosphate, Udaipur rock phosphate, and North Carolina rock phosphate [48]. In this study, transcriptome and HPLC analysis showed that formic acid and acetic acid played important roles in the improvement of available P fractions under NH_4_^+^ stress, and the concentrations of formic acid and acetic acid in the high-NH_4_^+^ group were generally higher, about 2.77 and 2.1 folds, respectively (Fig. 4; Table 3). It has been reported that acetate would accumulate when the glycolysis flux exceeded the catalytic capacity of the TCA cycle [49]. Therefore, high glycolysis rate might present in *B. aryabhattai* NM1-A2, which was responsible for the accumulation of acetic acid under NH_4_^+^ stress (Fig. 4). Furthermore, the PS process was affected by the properties of organic acids. Although formic acid and acetic acid were both mono-carboxylic acids, formic acid had a higher PS ability than acetic acid [50]. Isocitrate lyase was a key enzyme of the glyoxylate cycle that was the bypassed pathway of the TCA cycle [51]. As shown in Table 3, *ackA* was downregulated under NH_4_^+^ stress, which suggested that the transfer of energy metabolism to the glyoxylate cycle was inhibited. This might lead to a decrease in succinic acid concentration in the high-NH_4_^+^ group (Fig. 4).

## Conclusion

*B. aryabhattai* NM1-A2 was screened from marine mangrove sediments. The soluble phosphate concentration in culture supernatant of *B. aryabhattai* NM1-A2 was continuously improved with the increase of (NH_4_)_2_SO_4_ concentration. Integrated omics technology was used to reveal the effects of NH_4_^+^ stress on PS of *B. aryabhattai* NM1-A2 for the first time. The inhibitions of AMT and GS/GOGAT were helpful to prevent the waste of energy. The GDH pathway was the main approach for NH_4_^+^ assimilation, in which glycolysis and TCA cycle could provide abundant C skeletons. The secretion of protons, formic acid, and acetic acid played a key role in PS of *B. aryabhattai* NM1-A2 under NH_4_^+^ stress. This study demonstrated a scientific basis for the development of biofertilizers.

## Methods

### Samples and media

The sediment samples were isolated from the National Shankou Natural Reserve of Mangrove in Beihai, Guangxi, China (21°29′25.74′′N, 109°45′49.43′′E) using the methods described in a previous study [52].

The following media were used in this study: Luria–Bertani medium (LB) containing (g/L) tryptone (10 g), yeast extract (5 g) and NaCl (10 g); SRSM enrichment medium [53] containing (g/L) glucose (10 g), (NH_4_)_2_SO_4_ (0.5 g), NaCl (20 g), KCl (0.2 g), FeSO_4_·7H_2_O (0.004 g), MgSO_4_·7H_2_O (0.3 g), MnSO_4_·H_2_O (0.0045 g), yeast extract (0.5 g), Ca_3_(PO_4_)_2_ (5 g). The initial pH of the medium for both groups was adjusted to 7.0 by adding NaOH or HCl.

### Screening for PSMs

The sediment (5.0 g) was mixed with LB medium (10 mL), followed by shaking for 1 h in a biochemical incubator at 37 °C, 200 rpm. Then serial dilutions (10^0^–10^− 3^) were spread-plated onto LB medium plates. After incubation for 12 h at 37 °C, colonies were selected from the plates. Then, these isolates were purified by the streak plate method. Each isolate was added to the SRSM medium plates to verify its PS ability by the presence of a clear halo around colonies.

### Identification of the isolated strains

All strains were cultured in 100 mL Erlenmeyer flasks containing 50 mL LB at 37 °C with continuous rocking on a shaker at 200 rpm for 12 h. Total genomic DNA was extracted using a TIANamp bacterial DNA kit (TIANGEN) according to the instructions of the manufacturer. The quality of the extracted DNA was measured by 1% agarose gel electrophoresis and analyzed using a NanoDrop™ 2000 spectrophotometer [5] (Thermo Scientific, USA). The amplified products of 16 S rDNA were obtained with specific primers [21] (Table S1) under the following reaction conditions: 95 ℃, 5 min; [95 °C for 30 s; 54 °C for 30 s; and 72 °C for 1 min] × 35 cycles; and 72 °C, 7 min. The PCR products were verified by 1% agarose gel electrophoresis, and they were sequenced using the Sanger sequencing platform (BGI: https://www.bgi.com/). The nucleotide sequences of 16 S rDNA were compared with sequences in NCBI (http://www.ncbi.nlm.nih.gov/GenBank) to identify close phylogenetic relatives. And the 16 S rDNA phylogenetic tree was constructed by using the neighbor-joining method supported by 1,000 bootstrap replicates in MEGA 7.0 [54].

### Determination of the soluble phosphate concentration in culture supernatant

The seven strains were cultured on SRSM medium for 48, 72, 96, and 120 h, and then centrifuged at 6, 000 rpm for 5 min. Then, the concentration of soluble phosphate in culture supernatant was determined by molybdenum blue colorimetric method [21]. A separate SRSM medium inoculated with sterile double-distilled water served as the control.

For optimization purpose, *B. aryabhattai* NM1-A2 was cultured for 12 h on SRSM medium containing C sources (glucose, sucrose, starch, fructose, and maltose), N sources ((NH_4_)_2_SO_4_, NH_4_Cl, CO(NH_2_)_2_, and KNO_3_), C/N (5, 20, and 40), initial solution pH (5.0, 6.0, 7.0, 8.0, 9.0, and 10.0), NaCl concentrations (0%, 2%, 4%, 6%, 8%, and 10%) and (NH_4_)_2_SO_4_ concentrations (1-300 mM). Then the concentration of soluble phosphate in culture supernatant of *B. aryabhattai* NM1-A2 was detected by the same method as described above. The final solution pH was determined using a pH meter (PHS-3 C, China Sanxin).

### Organic acids production of *B. aryabhattai* NM1-A2 under NH_4_^+^ stress

The types and concentrations of organic acid in the supernatants of the low- and high-NH_4_^+^ groups were analyzed using HPLC method [6]. The following organic acids (i.e., formic acid, malic acid, lactic acid, acetic acid, citric acid, and succinic acid) were measured using an Alliance liquid chromatograph (Waters E2695, United States). The liquid chromatograph column was Poroshell120 SB-Aq 4.6×250 mm, 4 μm (Agilent Technologies Inc., America), with a column temperature of 25 ℃. The detection wavelength was 210 nm.

### Whole-genome sequencing and assembly of *B. aryabhattai* NM1-A2

The whole-genome sequencing and assembly were performed by PFOMIC Bioinformatics Company (Nanning, China) with the Illumina NovaSeq 6000 PE150, Oxford Nanopore Technologies PromethION [54]. The whole genome data of *B. aryabhattai* NM1-A2 have been deposited in NCBI GeneBank with the accession number PRJNA760884. The MinKNOW software was used for filtering the low-quality reads. Then, the filtered reads were assembled by using the Unicycler (0.4.8) software. Moreover, Pilon software was used to obtain more accurate genome. Finally, DIAMOND was used to align the predicted protein sequence with the Kyoto Encyclopedia of Genes and Genomes (KEGG) [55]. For the alignment of each protein sequence, the match with the highest score (default identity ≥ 30%) was used to select the functional annotation.

### Transcriptome sequencing analysis of *B. aryabhattai* NM1-A2

We analyzed the transcriptome of *B. aryabhattai* NM1-A2 in the low- and high-NH_4_^+^ groups to study the transcriptional expression changes between the N deficient and the NH_4_^+^-stressed environment. The RNA of *B. aryabhattai* NM1-A2 was extracted using the TRIzol method [56]. Qualified libraries were sequenced on the Illumina Novaseq sequencing platform using the paired-end sequencing method (PE150). FastQC_v0.11.3 was used for quality control of the raw sequencing data [57]. The filtered sequence was aligned with the rRNA database using Bowtie2 to remove the rRNA sequence. Qualified sequencing data were aligned to the *B. aryabhattai* NM1-A2 genome using Hisat2, and the count value and the Fragments Per Kilobase of transcript per Million mapped reads (FPKM) values of the genes were calculated using HTseq-count and AWK script. EdgeR was used to calculate the differentially expressed genes (DEGs) [*p* < 0.05 and |Log_2_(fold change) | > 1]. Then, Gene Ontology (GO) and KEGG databases were used for functional enrichment of DEGs [55].

### Verification experiment

RT-qPCR was used to validate the transcriptomic data. A two-step method was used for the RT-qPCR of key genes with specific primers (Table S2) [21]. *GAPDH* was used as a reference gene for the normalization. The expression levels of selected genes were evaluated using the 2^−ΔΔCt^ method.

### Statistical analyses

All experimental treatments were conducted in triplicate, and the results are expressed as the means ± standard error. Data were processed with SPSS 24 and Diamond software. The relevant query databases were KEGG and NCBI. The least significant difference test based on the analysis of variance model was used for multiple comparisons among treatments groups for soluble phosphate concentration or pH value. And two independent-sample t-test was used to determine differences in organic acid concentration between the low- and high-NH_4_^+^ group. All figures were made with GraphPad Prism 8.0.2 (263) and Adobe Illustrator (2020).

### Electronic supplementary material

Below is the link to the electronic supplementary material.

**Figure S1**: Chromatograms of six organic acids mixed standard solutions. The numbers 1, 2, 3, 4, 5 and 6 represent formic acid, malic acid, lactic acid, acetic acid, citric acid and succinic acid, respectively. **Figure S2**: Validation of key genes related to NH4+ assimilation and phosphate transport and metabolism by RT-qPCR. **Table S1** Primers used for the amplification of 16S DNA of seven PSMs screened in this study [1]. **Table S2** 16S rDNA comparison results of seven PSMs screened in this study. **Table S3** Chromatographic retention time, standard curve equation, and correlation coefficient of six organic acid mixed standard solutions. **Table S4** The KEGG annotation results of genes relevant to NH4+ assimilation and phosphate transport and metabolism in B. aryabhattai NM1-A2 [2]. **Table S5** Primers used in RT-qPCR.

## Data Availability

The datasets presented in this study can be found in online repositories. The whole genome sequences are available from the NCBI nucleotides database under accessions NZ_CP083269 − NZ_CP083272. The transcriptome data has been uploaded to the SRA database of the NCBI database, the accession numbers are SRX18707345 − SRX18707349.

## References

[1] Wang ZH, Zhang HH, Liu L, Li SJ, Xie JF, Xue X (2022). Screening of phosphate-solubilizingbacteria and their abilities of phosphorus solubilization and wheat growth promotion. BMC Microbiol.

[2] Reef R, Feller IC, Lovelock CE (2010). Nutrition of mangroves. Tree Physiol.

[3] Zeng QW, Ding XL, Wang JC, Han XJ, Iqbal HM, Bilal M (2022). Insight into soil nitrogen and phosphorus availability and agricultural sustainability by plant growth-promoting rhizobacteria. Environ Sci Pollut Res.

[4] Liu YQ, Wang YH, Kong WL, Liu WH, Xie XL, Wu XQ (2020). Identification, cloning and expression patterns of the genes related to phosphate solubilization in *Burkholderia multivorans* WS-FJ9 under different soluble phosphate levels. AMB Express.

[5] Zhang ZF, Nie SQ, Sang YM, Mo SM, Li JH, Kashif M (2022). Effects of *Spartina alterniflora* invasion on nitrogen fixation and phosphorus solubilization in a subtropical marine mangrove ecosystem. Microbiol spectr.

[6] Li LL, Chen RB, Zuo ZY, Lv ZS, Yang ZH, Mao W (2020). Evaluation and improvement of phosphate solubilization by an isolated bacterium *Pantoea agglomerans* ZB. World J Microbiol Biotechnol.

[7] Behera BC, Singdevsachan SK, Mishra RR, Dutta SK, Thatoi HN (2014). Diversity, mechanism and biotechnology of phosphate solubilising microorganism in mangrove—a review. Biocatal Agric Biotechnol.

[8] Alori ET, Glick BR, Babalola OO (2017). Microbial phosphorus solubilization and its potential for use in sustainable agriculture. Front Microbiol.

[9] Sharma SB, Sayyed RZ, Trivedi MH, Gobi TA (2013). Phosphate solubilizing microbes: sustainable approach for managing phosphorus deficiency in agricultural soils. Springerplus.

[10] Rawat P, Das S, Shankhdhar D, Shankhdhar SC (2021). Phosphate-solubilizing microorganisms: mechanism and their role in phosphate solubilization and uptake. J Soil Sci Plant Nutr.

[11] Ding YQ, Yi ZL, Fang Y, He SL, Li YM, He KZ (2021). Multi-omics reveal the efficient phosphate-solubilizing mechanism of bacteria on rocky soil. Front Microbiol.

[12] Scervino JM, Papinutti VL, Godoy MS, Rodriguez MA, Monica DI, Recchi M (2011). Medium pH, carbon and nitrogen concentrations modulate the phosphate solubilization efficiency of Penicillium purpurogenum through organic acid production. J Appl Microbiol.

[13] Sarikhani MR, Khoshru B, Greiner R (2019). Isolation and identification of temperature tolerant phosphate solubilizing bacteria as a potential microbial fertilizer. World J Microbiol Biotechnol.

[14] An R, Moe LA (2016). Regulation of pyrroloquinoline quinone-dependent glucose dehydrogenase activity in the model rhizosphere-dwelling bacterium *Pseudomonas putida* KT2440. Appl Environ Microbiol.

[15] Li BH, Li GJ, Kronzucker HJ, Baluska F, Shi WM (2014). Ammonium stress in *Arabidopsis*: signaling, genetic loci, and physiological targets. Trends Plant Sci.

[16] Zhu XF, Dong XY, Wu Q, Shen RF (2019). Ammonium regulates Fe deficiency responses by enhancing nitric oxide signaling in *Arabidopsis thaliana*. Planta.

[17] Emma FC, Gemma C, Pilar GA (2012). Ammonium enhances resistance to salinity stress in citrus plants. J Plant Physiol.

[18] Ma SN, Wang HJ, Wang HZ, Li Y, Liu M, Liang XM (2018). High ammonium loading can increase alkaline phosphatase activity and promote sediment phosphorus release: a two-month mesocosm experiment. Water Res.

[19] Hachiya T, Inaba J, Wakazaki M, Sato M, Toyooka K, Miyagi A (2021). Excessive ammonium assimilation by plastidic glutamine synthetase causes ammonium toxicity in *Arabidopsis thaliana*. Nat Commun.

[20] Hachiya T, Watanabe CK, Fujimoto M, Ishikawa T, Takahara K, Kawai-Yamada M (2012). Nitrate addition alleviates ammonium toxicity without lessening ammonium accumulation, organic acid depletion and inorganic cation depletion in *Arabidopsis thaliana* shoots. Plant Cell Physiol.

[21] Aliyat FZ, Maldani M, Guilli ME, Nassiri L, Ibijbijen J (2022). Phosphate-solubilizing bacteria isolated from phosphate solid sludge and their ability to solubilize three inorganic phosphate forms: aalcium, iron, and aluminum phosphates. Microorganisms.

[22] Tian XP, Fang Y, Jin YL, Yi ZL, Li JM, Du AP (2021). Ammonium detoxification mechanism of ammonium-tolerant duckweed (*Landoltia punctata*) revealed by carbon and nitrogen metabolism under ammonium stress. Environ Pollut.

[23] Meng S, Su L, Li YM, Wang YJ, Zhang CX, Zhao Z (2016). Nitrate and ammonium contribute to the distinct nitrogen metabolism of *Populus simonii* during moderate salt stress. PLoS ONE.

[24] Luz DE, Nepomuceno RS, Spira B, Ferreira RC (2012). The pst system of *Streptococcus* mutans is important for phosphate transport and adhesion to abiotic surfaces. Mol Oral Microbiol.

[25] Grafe M, Goers M, Tucher SV, Baum C, Zimmer D, Leinweber P (2018). Bacterial potentials for uptake, solubilization and mineralization of extracellular phosphorus in agricultural soils are highly stable under different fertilization regimes. Environ Microbiol Rep.

[26] Yuan ZC, Zaheer R, Finan TM (2006). Regulation and properties of PstSCAB, a high-affinity, high-velocity phosphate transport system of *Sinorhizobium meliloti*. J Bacteriol.

[27] Luttmann D, Gopel Y, Gorke B (2012). The phosphotransferase protein EIIA^Ntr^ modulates the phosphate starvation response through interaction with histidine kinase PhoR in *Escherichia coli*. Mol Microbiol.

[28] Gosztolai A, Schumacher J, Behrends V, Bundy JG, Heydenreich F, Bennett MH (2017). GlnK facilitates the dynamic regulation of bacterial nitrogen assimilation. Biophys J.

[29] Smith DP, Thrash JC, Nicora CD, Lipton MS, Burnum-Johnson KE, Carini P (2013). Proteomic and transcriptomic analyses of “*Candidatus Pelagibacter* ubique” describe the first P_II_-independent response to nitrogen limitation in a free-living *Alphaproteobacterium*. mBio.

[30] Bernard SM, Habash DZ (2009). The importance of cytosolic glutamine synthetase in nitrogen assimilation and recycling. New Phytol.

[31] Wang F, Wang Q, Yu QG, Ye J, Gao J, Liu HT (2022). Is the NH_4_^+^-induced growth inhibition caused by the NH_4_^+^ form of the nitrogen source or by soil acidification?. Front Plant Sci.

[32] Martin P, Dyhrman ST, Lomas MW, Poulton NJ, Van Mooy BAS (2014). Accumulation and enhanced cycling of polyphosphate by Sargasso Sea plankton in response to low phosphorus. Proc Nat Acad Sci.

[33] Mirra B, Carvalho K, Curitiba B, Ribeiro L, Moraes J, da Silva JR (2019). Inorganic pyrophosphatase from the red flour beetle (*Tribolium castaneum*) modulates mitochondrial polyphosphate metabolism. Arch Insect Biochem Physiol.

[34] Wei YQ, Zhao Y, Shi MZ, Cao ZY, Lu Q, Yang TX (2018). Effect of organic acids production and bacterial community on the possible mechanism of phosphorus solubilization during composting with enriched phosphate-solubilizing bacteria inoculation. Bioresour Technol.

[35] Bhattacharyya C, Bakshi U, Mallick I, Mukherji S, Bera B, Ghosh A (2017). Genome-guided insights into the plant growth promotion capabilities of the physiologically versatile *Bacillus aryabhattai* strain AB211. Front Microbiol.

[36] Nelofer R, Syed Q, Nadeem M, Bashir F, Mazhar S, Hassan A (2016). Isolation of phosphorus-solubilizing fungus from soil to supplement biofertilizer. Arab J Sci Eng.

[37] Chen HY, Chen YN, Wang HY, Liu ZT, Frommer WB, Ho CH (2020). Feedback inhibition of AMT1 NH_4_^+^-transporters mediated by CIPK15 kinase. BMC Biol.

[38] Sayavedra-Soto L, Ferrell R, Dobie M, Mellbye B, Chaplen F, Buchanan A (2015). *Nitrobacter winogradskyi* transcriptomic response to low and high ammonium concentrations. FEMS Microbiol Lett.

[39] Stein LY, Campbell MA, Klotz MG (2013). Energy-mediated vs. ammonium-regulated gene expression in the obligate ammonia-oxidizing bacterium, *Nitrosococcus oceani*. Front Microbiol.

[40] Hess DC, Lu W, Rabinowitz JD, Botstein D (2006). Ammonium toxicity and potassium limitation in yeast. PLoS Biol.

[41] Kim M, Zhang ZG, Okano H, Yan DL, Groisman A, Hwa T (2012). Need-based activation of ammonium uptake in *Escherichia coli*. Mol Syst biol.

[42] Sathee L, Jha SK, Rajput OS, Singh D, Kumar S, Kumar A (2021). Expression dynamics of genes encoding nitrate and ammonium assimilation enzymes in rice genotypes exposed to reproductive stage salinity stress. Plant Physiol Biochem.

[43] Burkovski A (2003). Ammonium assimilation and nitrogen control in *Corynebacterium glutamicum* and its relatives: an example for new regulatory mechanisms in actinomycetes. FEMS Microbiol Rev.

[44] Hodges M (2002). Enzyme redundancy and the importance of 2-oxoglutarate in plant ammonium assimilation. J Exp Bot.

[45] Gunka K, Commichau FM (2012). Control of glutamate homeostasis in *Bacillus subtilis*: a complex interplay between ammonium assimilation, glutamate biosynthesis and degradation. Mol Microbiol.

[46] Rubio PJ, Godoy MS, Monica IF, Pettinari MJ, Godeas AM, Scervino JM (2016). Carbon and nitrogen sources influence tricalcium phosphate solubilization and extracellular phosphatase activity by *Talaromyces flavus*. Curr Microbiol.

[47] Wang SB, Li Y, Zhang J, Wang XY, Hong JP, Qiu C (2022). Transcriptome profiling analysis of phosphate-solubilizing mechanism of Pseudomonas strain W134. Microorganisms.

[48] Gulati A, Sharma N, Vyas P, Sood S, Rahi P, Pathania V (2010). Organic acid production and plant growth promotion as a function of phosphate solubilization by *Acinetobacter rhizosphaerae* strain BIHB 723 isolated from the cold deserts of the trans-himalayas. Arch Microbiol.

[49] Chiang CJ, Hu RC, Huang ZC, Chao YP (2021). Production of succinic acid from amino acids in *Escherichia coli*. J Agric Food Chem.

[50] Basak BB (2019). Phosphorus release by low molecular weight organic acids from low-grade indian rock phosphate. Waste Biomass Valorization.

[51] Yuenyong W, Sirikantaramas S, Qu LJ, Buaboocha T (2019). Isocitrate lyase plays important roles in plant salt tolerance. BMC Plant Biol.

[52] Nie SQ, Zhang ZF, Mo SM, Li JH, He S, Kashif M (2021). Desulfobacterales stimulates nitrate reduction in the mangrove ecosystem of a subtropical gulf. Sci Total Environ.

[53] Vazquez P, Holguin G, Puente ME, Lopez-Cortes A, Bashan Y (2000). Phosphate-solubilizing microorganisms associated with the rhizosphere of mangroves in a semiarid coastal lagoon. Biol Fertil Soils.

[54] Kashif M, Lu ZM, Sang YM, Yan B, Shah SJ, Khan S (2022). Whole-genome and transcriptome sequencing-based characterization of *Bacillus Cereus* NR1 from subtropical marine mangrove and its potential role in sulfur metabolism. Front Microbiol.

[55] Kanehisa M, Furumichi M, Sato Y, Kawashima M, Ishiguro-Watanabe M (2023). KEGG for taxonomy-based analysis of pathways and genomes. Nucleic Acids Res.

[56] Kashif M, Sang YM, Mo SM, Rehman SE, Khan S, Khan MR (2023). Deciphering the biodesulfurization pathway employing marine mangrove *Bacillus aryabhattai* strain NM1-A2 according to whole genome sequencing and transcriptome analyses. Genomics.

[57] Chen SF, Zhou YQ, Chen YR, Gu J (2018). Fastp: an ultra-fast all-in-one FASTQ preprocessor. Bioinformatics.

