# Supplementary material for: Effect of ammonium stress on phosphorus solubilization of a novel marine mangrove microorganism Bacillus aryabhattai NM1-A2 as revealed by integrated omics analysis
Source: BMC Genomics. 2023 Sep 18;24:550. doi: 10.1186/s12864-023-09559-z (PMC10506230; doi:10.1186/s12864-023-09559-z)
Supplement: Supplementary file 1 — Figure S1: Chromatograms of six organic acids mixed standard solutions. The numbers 1, 2, 3, 4, 5 and 6 represent formic acid, malic acid, lactic acid, acetic acid, citric acid and succinic acid, respectively. Figure S2: Validation of key genes related to NH4+ assimilation and phosphate transport and metabolism by RT-qPCR. Table S1 Primers used for the amplification of 16S DNA of seven PSMs screened in this study [1]. Table S2 16S rDNA comparison results of seven PSMs screened in this study. Table S3 Chromatographic retention time, standard curve equation, and correlation coefficient of six organic acid mixed standard solutions. Table S4 The KEGG annotation results of genes relevant to NH4+ assimilation and phosphate transport and metabolism in B. aryabhattai NM1-A2 [2]. Table S5 Primers used in RT-qPCR. [file 12864_2023_9559_MOESM1_ESM.docx]

**ADDITIONAL FILE 1**

**Effect of ammonium stress on** **phosphorus solubilization of a novel marine mangrove microorganism** ***Bacillus aryabhattai* NM1-A2 as revealed by** **integrated omics analysis**

Zhaomei Lu ^1, 2, 3, †^ , Sheng He ^4,^ ^†^ , Muhammad Kashif ^1, 2^, Zufan Zhang ^1^, Shuming Mo ^1^, Guijiao Su ^1^, Linfang Du ^3, *^ and Chengjian Jiang ^1, 2, *^

^1^ State Key Laboratory for Conservation and Utilization of Subtropical Agro-bioresources, Guangxi Research Center for Microbial and Enzyme Engineering Technology, College of Life Science and Technology, Guangxi University, Nanning 530004, China.

^2^ Guangxi Key Laboratory for Green Processing of Sugar Resources, College of Biological and Chemical Engineering, Guangxi University of Science and Technology, Liuzhou 545006, China.

^3^ Key Laboratory of Bio-resources and Eco-environment of the Ministry of Education, College of Life Sciences, Sichuan University, Chengdu 610064, China.

^4^ Guangxi Key Laboratory of Birth Defects Research and Prevention, Guangxi Key Laboratory of Reproductive Health and Birth Defect prevention, Guangxi Zhuang Autonomous Region Women and Children Health Care Hospital, Nanning 530033, China.

**^*^ Corresponding Author:**

Tel.: +86-28-85415008; Fax: +86-28-85415300

E-mail: dulinfang@scu.edu.cn (LD)

Tel.: +86-771-3239403; Fax: +86-771-3239403.

E-mail: jiangcj@gxu.edu.cn (CJ)
^†^ : Zhaomei Lu and Sheng He contributed equally to this article.


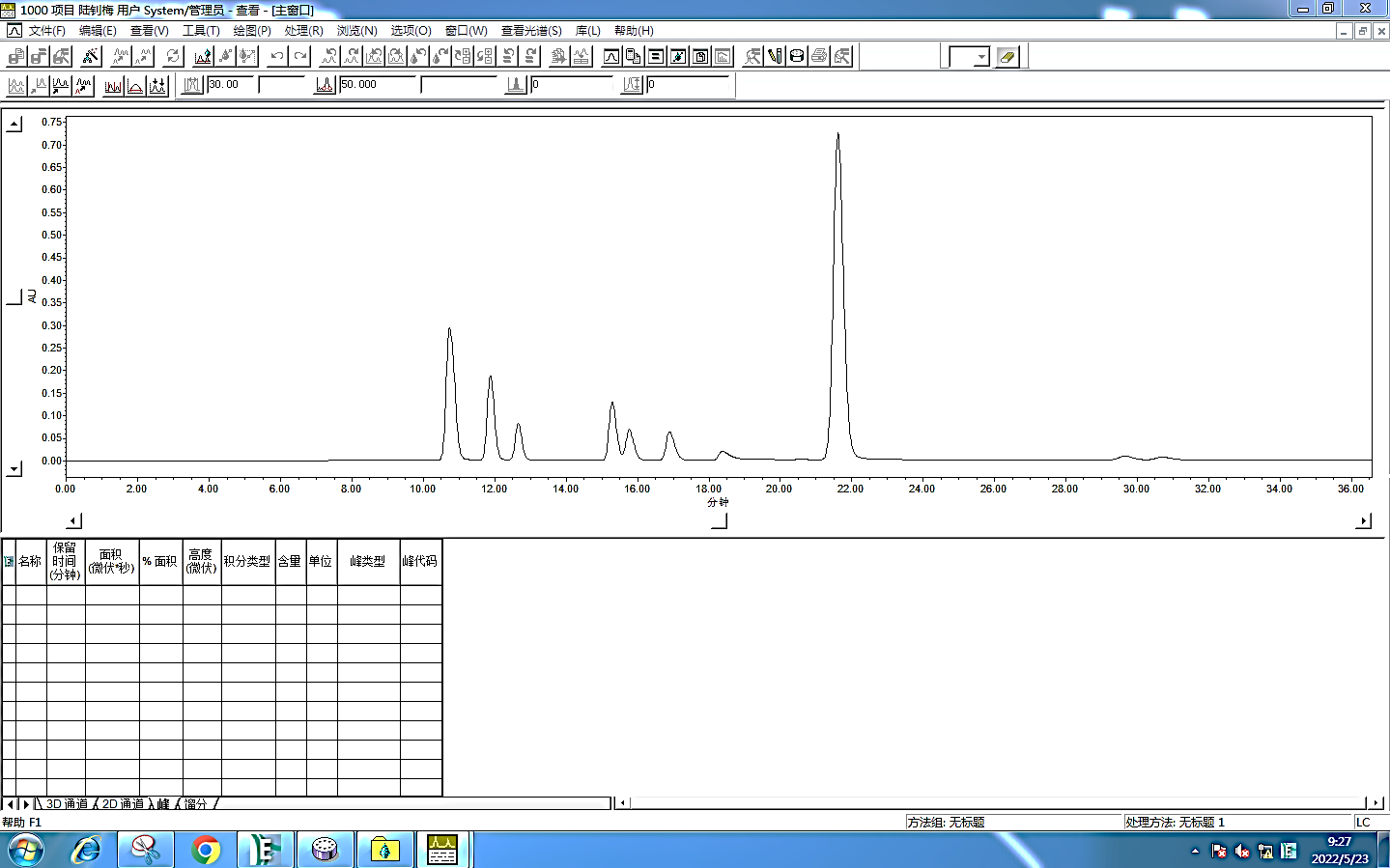


**6**

**3**

**4**

**5**

**2**

**1**

**Figure S1:** Chromatograms of six organic acids mixed standard solutions. The numbers 1, 2, 3, 4, 5 and 6 represent formic acid, malic acid, lactic acid, acetic acid, citric acid and succinic acid, respectively.

**
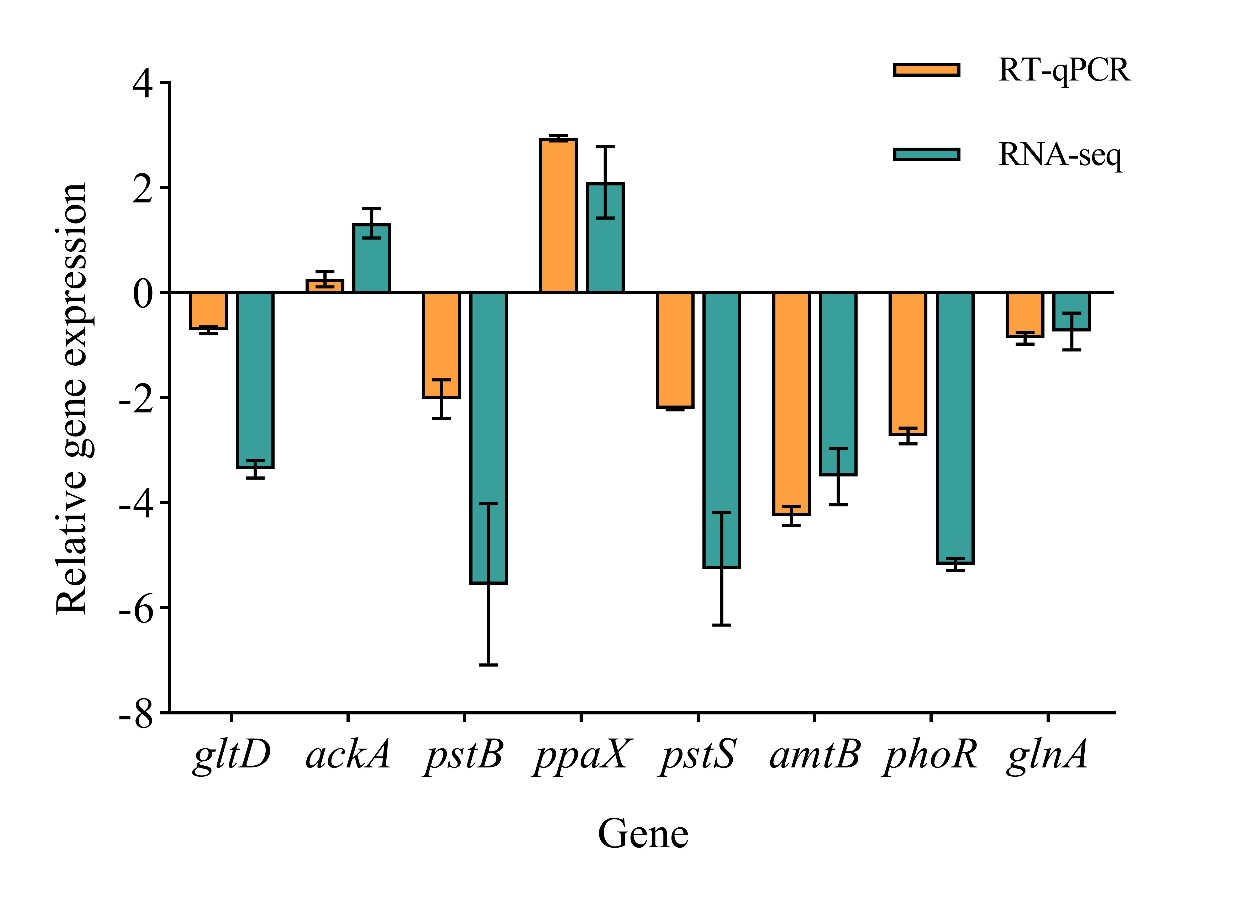
**

**Figure S2:** Validation of key genes related to NH_4_^+^ assimilation and phosphate transport and metabolism by RT-qPCR.

**Table S1** Primers used for the amplification of 16S DNA of seven PSMs screened in this study [1].

| Gene | Primer | Sequence（5’-3’） | Amplicon size (bp) | Annealing temperature (℃) |
| --- | --- | --- | --- | --- |
| 16S rDNA | 27 F | AGAGTTTGATCCTGGCTCAG | 1465 | 54 |
| 16S rDNA | 1492 R | ACGGTTACCTTGTTACGACTT | 1465 | 54 |

**Table S2** 16S rDNA comparison results of seven PSMs screened in this study.

| Strain | Length | Description | Taxonomy | Cover (%) | Identity (%) | Accession |
| --- | --- | --- | --- | --- | --- | --- |
| NM1-A2 | 1414 | *Priestia aryabhattai* B8W22 | *Priestia* | 100 | 100 | NR_115953.1 |
| HM2 | 1469 | *Metabacillus niabensis* strain 4T19 | *Metabacillus* | 99 | 98.7 | NR_043334.1 |
| HM3 | 1417 | *Priestia aryabhattai* B8W22 | *Priestia* | 100 | 100 | NR_115953.1 |
| HM4 | 1412 | *Bacillus haynesii* strain NRRL B-41327 | *Bacillus* | 100 | 99.65 | NR_157609.1 |
| HM5 | 1413 | *Bacillus licheniformis* strain DSM 13 | *Bacillus* | 99 | 99.93 | NR_118996.1 |
| HM6 | 1469 | *Bacillus licheniformis* strain DSM 13 | *Bacillus* | 98 | 98.08 | NR_118996.1 |
| HM7 | 1399 | *Bacillus velezensis* strain FZB42 | *Bacillus* | 100 | 99.93 | NR_075005.2 |

**Table S3** Chromatographic retention time, standard curve equation, and correlation coefficient of six organic acid mixed standard solutions.

| Organic acid | Retention time | Standard curve equation | Correlation coefficient |
| --- | --- | --- | --- |
| Formic acid | 11.88 | y=2246.1x+5513.9 | 0.9995 |
| Malic acid | 12.659 | y=1116.8x-4643.9 | 0.9997 |
| Lactic acid | 15.291 | y=1922x+2552.3 | 0.9990 |
| Acetic acid | 15.769 | y=1033.4x+7403.6 | 0.9985 |
| Citric acid | 16.9 | y=1085.4x-1357.1 | 0.9997 |
| Succinic acid | 21.617 | y=14701x+13304 | 0.9994 |

**Table S4** The KEGG annotation results of genes relevant to NH_4_^+^ assimilation and phosphate transport and metabolism in *B. aryabhattai* NM1-A2 [2].

| Pathway | ORF name | Gene symbol | Function | accession numbers |
| --- | --- | --- | --- | --- |
| NH_4_^+^ assimilation | K8Z47_RS03380 | *amt* | Ammonium transporter | K03320 |
|  | K8Z47_RS11985 | *gdhA* | Glutamate dehydrogenase | K00260 |
|  | K8Z47_RS20175 | *glnA* | Glutamine synthetase, type I | K01915 |
|  | K8Z47_RS21810 | *gltD* | Glutamate synthase [NADPH] small chain | K00266 |
|  | K8Z47_RS10260 | *gltB* | Glutamate synthase [NADPH] large chain | K00265 |
| Phosphate transport and metabolism | K8Z47_RS25960 | *phoR* | Phosphate regulon sensor histidine kinase PhoR | K07636 |
|  | K8Z47_RS06210 | *phoB* | Phosphate regulon response regulator PhoB | K07657 |
|  | K8Z47_RS22250 | *phoU* | Phosphate transport system regulatory protein PhoU | K02039 |
|  | K8Z47_RS22810 | *pstS* | Phosphate-binding protein PstS 1 precursor | K02040 |
|  | K8Z47_RS22805 | *pstC* | Phosphate ABC transporter, permease protein PstC | K02037 |
|  | K8Z47_RS22800 | *pstA* | Phosphate transport system permease protein PstA | K02038 |
|  | K8Z47_RS22255 | *pstB* | Phosphate ABC transporter, ATP-binding protein PstB | K02040 |
|  | K8Z47_RS25095 | *ppaX* | Pyrophosphatase PpaX | K06019 |
|  | K8Z47_RS23660 | *ackA* | Acetate kinase | K00925 |
|  | K8Z47_RS24905 | *fdhD* | Formate dehydrogenase accessory protein | K02379 |
|  | K8Z47_RS23540 | *icd* | Isocitrate dehydrogenase [NADP] | K00031 |
|  | K8Z47_RS03260 | *aceA* | Isocitrate lyase | K01637 |
|  | K8Z47_RS01990 | *fumA* | Fumarate hydratase, class I | K01676 |
|  | K8Z47_RS23535 | *mdh* | Malate dehydrogenase | K00024 |
|  | K8Z47_RS14710 | *glnK* | P_II_ family nitrogen regulator | K07717 |
|  | K8Z47_RS03375 | *glnB* | P_II_ family nitrogen regulator | K04751 |

**Table S5** Primers used in RT-qPCR.

| Gene | Forward Primer（5’-3’） | Reverse Primer（5’-3’） |
| --- | --- | --- |
| *amt* | GGTGCCTTAGCTGGACTTGT | CTGCAATCAGCCCAATGACG |
| *gltD* | ACGATTCCAGTCGATGCTGT | ACTCCGTCTTCATGGTTCAGC |
| *glnA* | GACTTAGCGCCAACTGACCT | ATCTCGTGTTGTCCAGGAGC |
| *pstS* | CAACGTAATCAACCGTCCAGC | AGCTGTACCAACGCTGTCAT |
| *pstB* | AGGAGCAGCTATTTGGGACG | GCAGCGCGCAATACAGATAC |
| *ackA* | TCGGTTCTTATGCTGCTCGT | CTAAACCACGAAGAACGCGAG |
| *ppaX* | TGGCTGCACGAGCAGAATTA | AGGATGCGGCTTTGCATTTG |
| *phoR* | GGCTGGCTAAGGATCCACAT | ATGAGGCTCGTCGTGTTGTC |
| *GAPDH* | GGTTACAGCGAAGAGCCACT | TCCGCTCTCGTTATCGTACC |

**References**

1. Aliyat FZ, Maldani M, Guilli ME, Nassiri L, Ibijbijen J. Phosphate-solubilizing bacteria isolated from phosphate solid sludge and their ability to solubilize three inorganic phosphate forms: calcium, iron, and aluminum phosphates. Microorganisms. 2022; 10(5): 980.

2. Kanehisa M, Furumichi M, Sato Y, Kawashima M, Ishiguro-Watanabe M. KEGG for taxonomy-based analysis of pathways and genomes. Nucleic Acids Res. 2023; 51(D1): D587-D592.
